# Ocular surface cooling rate associated with tear film characteristics and the maximum interblink period

**DOI:** 10.1038/s41598-021-94568-9

**Published:** 2021-07-22

**Authors:** Jennifer E. Ding, Young Hyun Kim, Sarah M. Yi, Andrew D. Graham, Wing Li, Meng C. Lin

**Affiliations:** 1 Clinical Research Center, School of Optometry, University of California, Berkeley, 360 Minor Hall, Berkeley, CA 94720-2020 USA; 2 Vision Science Graduate Group, University of California, Berkeley, CA 94720 USA; 3 Chemical and Biomolecular Engineering Department, University of California, Berkeley, CA 94720 USA

**Keywords:** Medical research, Signs and symptoms

## Abstract

The surface of the human eye is covered with a protective tear film that refreshes with each blink. Natural blinking occurs involuntarily, but one can also voluntarily blink or refrain from blinking. The maximum time one can refrain from blinking until the onset of discomfort is the maximum interblink period (MIBP). During the interblink period the tear film evaporates and thins from the ocular surface. Infrared thermography provides a non-invasive measure of the ocular surface temperature (OST). Due to evaporation, ocular surface cooling (OSC) generally occurs when the eyes are open and exposed to the environment. The purpose of our study was to investigate the effect of OSC rate on the MIBP, and to investigate the association of the MIBP with tear film characteristics in subjects who do and do not exhibit OSC. The MIBP was measured simultaneously with OST over time. Non-invasive tear breakup time, tear meniscus height, tear lipid layer thickness, and Schirmer I test strip wetted lengths were measured on a day prior to the thermography visit. Subjects were divided into cooling and non-cooling groups based on OSC rate, and demographic and tear film characteristics were tested for inter-group differences. A faster OSC rate was associated with an exponentially shorter duration of the MIBP overall and within the cooling group alone. Faster non-invasive tear breakup time was significantly associated with a shorter MIBP in both groups. These results suggest that tear film evaporation initiates a pathway that results in the onset of ocular discomfort and the stimulus to blinking. The presence of a subset of subjects with no or minimal OSC who nevertheless have a short MIBP indicates that evaporative cooling is not the only mechanism responsible for the onset of ocular discomfort.

## Introduction

The human tear film plays an important role in ocular surface protection, lubrication, and stability of vision^1^. It is commonly accepted that tear film thinning occurs when the eyes are open, primarily due to tear evaporation^1–4^. Blinking introduces fresh tears, redistributes existing tears, and facilitates tear drainage to create a smooth refractive ocular surface. Natural, spontaneous blinks occur on average 14 to 22 times per minute^5–7^. Delay of blinking places evaporative stress on the tear film due to prolonged environmental exposure, leading to tear film thinning and breakup^3^. Evaporative tear film thinning can be assessed by thermographic imaging of the ocular surface during blink refrainment, and quantified as the ocular surface cooling (OSC) rate^2,8^.

Subjects can refrain from blinking generally until the onset of ocular discomfort, thought to be due in part to increased osmolarity in localized regions as the tear film thins and salts concentrate, stimulating afferent nerves^9–11^. Palaruku utilized optical coherence tomography to assess the effects of blinking on tear dynamics and found delay of blinking for as long as possible to be associated with elevated upper tear meniscus height, area, and volume; greater lower tear meniscus volume; and increased ocular surface tear volume due to increased reflex tearing and a potential decrease in tear drainage^12^. Reflex tearing is a sign of ocular discomfort and the composition of reflex tears differs from that of basal tears, with reflex tears exhibiting a lower pH and increased levels of free cholesterols and phospholipids^13,14^. An alternate method to assess open-eye tear dynamics without altering tear composition is by instructing subjects to refrain from blinking only until the onset of ocular discomfort (the maximum interblink period, MIBP)^2,8^. This method allows one to compare time to onset of ocular discomfort as evidenced by the length of the MIBP to the OSC rate and other tear film characteristics while minimizing reflex tearing.

Commercially available and novel instruments have been developed to assess tear film evaporation and its association with ocular dryness^1,15^. Ocular surface temperature (OST) during contact lens wear was first measured by Hill and Leighton using a modified scleral contact lens with attached contact probe in 1965^16^. Shortly thereafter Mapstone evaluated corneal surface temperature using a thermographic camera^17,18^. In 2015 Li et al. used an infrared thermographic video camera to study the relationship between ocular surface cooling rate and tear film thinning^2^. Li et al. concluded that a higher rate of ocular surface cooling was associated with faster fluorescein tear breakup formation, and that localized regions of cooling are detectable prior to tear breakup in those regions. OST decreases during the interblink period due to evaporative cooling as the tear aqueous evaporates^19,20^. The relationship observed between OST and fluorescein tear breakup suggests that infrared thermography can be used to indirectly assess tear evaporation and associated tear film instability.

The purpose of this study was to examine the relationship between OSC as an indication of tear evaporation^2^, and onset of ocular discomfort as represented by the MIBP. We hypothesize that a higher rate of OSC will be associated with a shorter MIBP. The association of the two would suggest that faster evaporation of the tears leads to a quicker thinning of the tear film, localized regions of hyperosmolarity, activation of corneal nerves and onset of ocular discomfort.

## Materials and methods

### Subjects

Subjects of at least 18 years of age with an oculo-visual examination within the previous two years were eligible to participate. Both contact lens wearers and non-contact lens wearers were eligible. Subjects were required to discontinue contact lens wear, eye drops and allergy medications 24 h prior to the measurement visit, and to cease use of makeup, lotions, or perfumes on the face the evening before the study visit. Active participation in any other clinical research study, use of antibiotics for active eye-related infections, use of anti-inflammatory medications for eye-related inflammation, or history of ocular surgery (e.g., eyelid or refractive surgery) within the previous 6 months excluded potential subjects from participation. Females pregnant, nursing, lactating, or planning pregnancy were also excluded. Informed consent was obtained from all subjects after a detailed explanation of the goals, procedures, risks, and potential benefits of the study. This study was approved by the University of California, Berkeley Committee for the Protection of Human Subjects and observed the tenets of the Declaration of Helsinki.

### Procedures

Enrolled subjects completed detailed medical and ocular history questionnaires. Subjects then completed the Pain Sensitivity Questionnaire (PSQ)^21,22^, the Berkeley Dry Eye Flow Chart (DEFC)^23^, and the Contact Lens Dry Eye Questionnaire (CLDEQ-8)^24^ in a pre-determined randomized order to assess pain sensitivity and ocular dryness, as well as to categorize contact lens wearing subjects as symptomatic or asymptomatic based on Young et al.’s criteria^25^. A clinician then conducted an ocular surface examination with a slit lamp under white light, and performed the following measurements: tear lipid layer thickness and coefficient of variation with the LipiView ocular surface interferometer (TearScience, Morrisville, NC, USA), tear meniscus height (TMH) with the Oculus Keratograph 5 M (Oculus Inc., Arlington, WA, USA), non-invasive tear breakup time (NITBUT) with the Medmont E300 corneal topographer (Medmont International PTY LTD, Nunawading, Australia), and closed-eye Schirmer I test strip wetted length (Merck & Co. Inc., Summit, NJ, USA). TMH and NITBUT were each measured three times and averaged. The Schirmer I test was administered without anesthetics.

**Figure 1:**
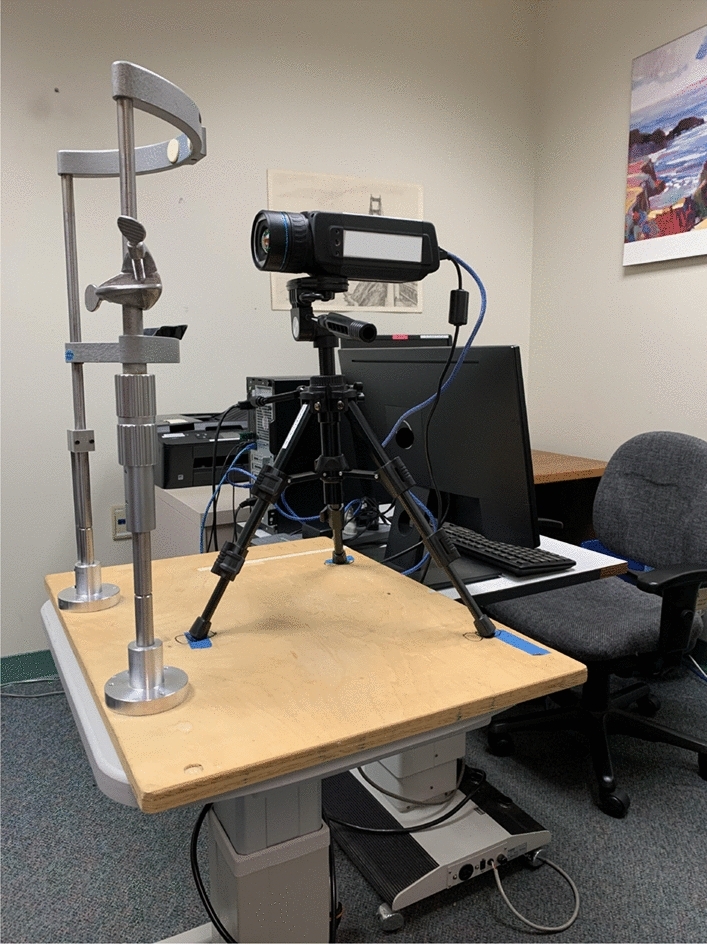
FLIR thermographic camera setup.

**Figure 2:**
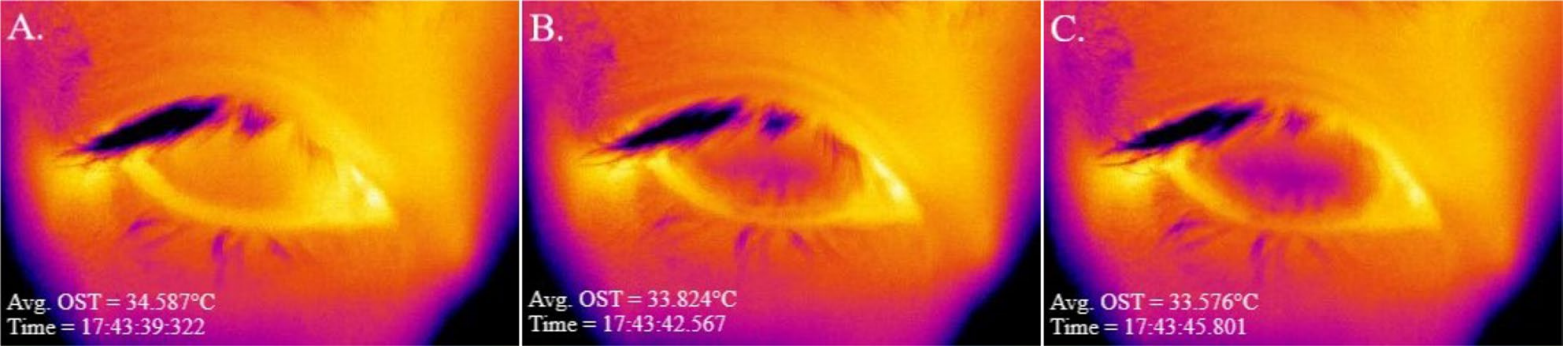
A series of screen captures of an OST video recording (**A**) immediately upon full eyelid opening, (**B**) the middle of the interblink period, and (**C**) the last frame before the start of eyelid closure. Darker (purple) region indicates area of OSC.

On a separate day, a minimum of 24 h and a maximum of 2 weeks following the initial visit, OST was measured concurrently with the MIBP with a commercially available infrared thermographic camera (Fig. 1), the FLIR A655sc (FLIR Systems Inc., Wilsonville, OR, USA). The subject was positioned in a mounted forehead and chin rest apparatus to maintain stable head position throughout the recording. The tripod-mounted thermographic camera lens was positioned approximately 6 cm directly in front of the apparatus. The investigator then adjusted the camera focus to the cornea of the subject’s right eye while providing instructions for fixation during the video recording. Subjects then acclimated to the ambient temperature and humidity in the examination room with closed eyes for two minutes. Upon eye opening, subjects focused on the center of the infrared camera lens and held their eyes open for as long as comfortably possible while the investigator recorded the thermographic video of the ocular surface (Fig. 2). The MIBP was calculated from the video recording time stamps of the first frame after full eye opening and the last frame before the start of a blink or eyelid closure. For the single subject who was able to keep the eyes open for > 100 s comfortably without reflex tearing, the MIBP was truncated at 100 s for statistical analysis.

OST readings were generated using the FLIR ResearchIR 4.40.8.28 Software (FLIR Systems Inc., Wilsonville, OR, USA. www.FLIR.com) and MATLAB R2018b (The MathWorks Inc., Natick, MA, USA. www.mathworks.com). Once a full MIBP was captured on thermographic video, a MATLAB script was used to define a best-fit octagon to the corneal area of interest in the first full frame after eye opening, and to then calculate the average temperature of all pixels in this region of interest frame by frame throughout the interblink period. The timestamps for each video frame were converted to elapsed time in seconds, and the frame-by-frame mean OST and the corresponding elapsed times were exported along with the length of the MIBP for statistical analysis.

### Statistical analysis

After obtaining the OST frame by frame for each subject from the infrared thermography video, a linear model of OST over time during the MIBP was fit to estimate the OSC rate^2^. An exponential model was then fit to examine the relationship across all subjects between OSC rate and MIBP length. Subjects were then stratified into two groups: subjects who exhibited a decrease in OST during the MIBP and subjects whose ocular surfaces did not appreciably drop in temperature during the MIBP. Details regarding the stratification criteria are given in the Results section. These two groups were then compared using simple linear models for differences in sex, ethnicity, symptom scores, Schirmer I test strip wetted lengths, NITBUT, TMH, and lipid layer mean thickness and coefficient of variability.

## Results

**Figure 3:**
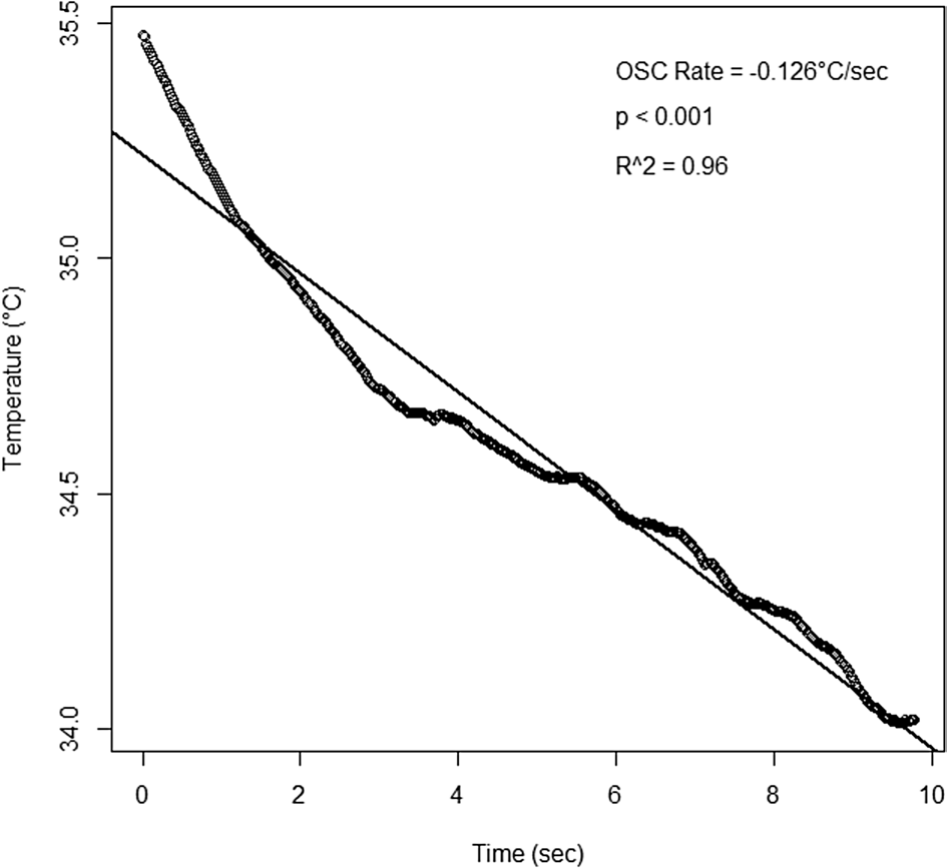
Change in ocular surface temperature over time to obtain the OSC rate for each subject.

Seventy-seven subjects (62 female, 15 male) completed the study. Their ages ranged from 18 to 35 years and the mean (SD) age was 23.1 (4.0) years. Forty-eight subjects were Asian and twenty-nine subjects were non-Asian. The OSC rate for each subject was obtained from the linear fit of OST over the MIBP. Figure 3 provides an example of a linear fit for a single subject. The grand mean (SD) OSC rate was − 0.063 (0.059) °C/s (the negative value indicates a reduction in temperature or cooling of the ocular surface). The grand mean (SD) MIBP was 14.47 (16.25) s. Across all subjects, the MIBP was significantly related to the OSC rate (*p* < 0.001), and the best fitting model was exponential:

1 $${\text{MIBP}} = {\text{e}}^{{({2}.{7481} + {6}.{3169}*{\text{OSCRATE}})}}$$

**Figure 4:**
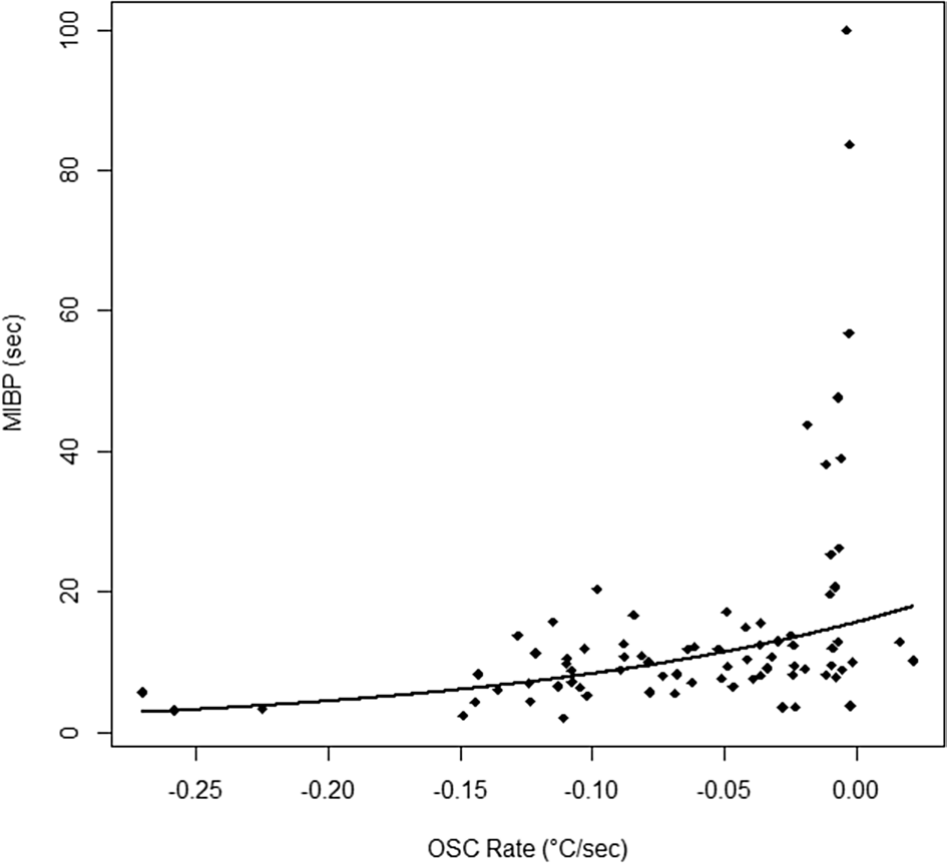
Exponential model of the MIBP as a function of OSC rate across all subjects.

Figure 4 depicts the exponential decrease in the MIBP with faster OSC rate across all subjects. As estimated by the model, the minimum observed OSC rate (i.e., the fastest cooling) of − 0.270 °C/s was associated with a MIBP of 2.83 s and the maximum OSC rate, which appeared as a slight OST warming of + 0.022 °C/s (see Discussion), was associated with a MIBP of 17.90 s.

### Ocular surface cooling sub-groups

**Figure 5:**
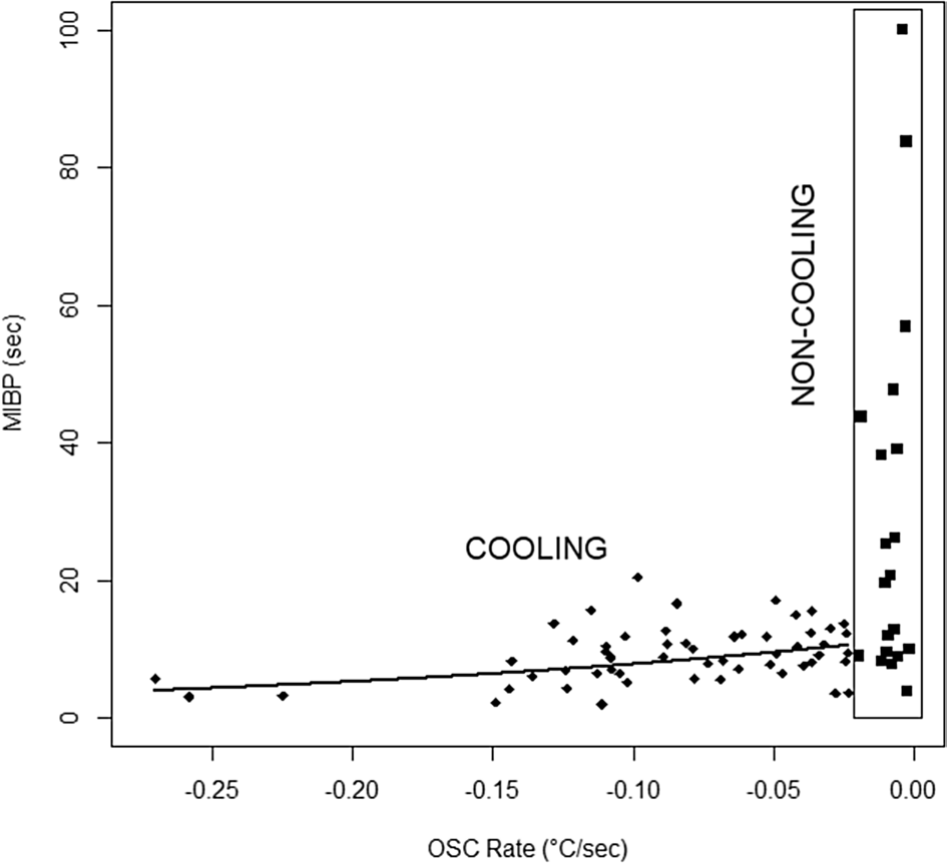
Stratification of subjects into faster OSC and minimal-to-no OSC groups.

Subjects were then divided into two groups: those who exhibited cooling of the ocular surface during the MIBP and those who exhibited minimal-to-no OSC (Fig. 5). The 77 subjects were stratified based on an OSC rate threshold of − 0.021 °C/s, with 55 subjects exhibiting lower rates (i.e., faster cooling) than the threshold and 22 subjects exhibiting minimal-to-no OSC based on this threshold. The threshold for minimal-to-no OSC was defined as having slower cooling than > 1 standard deviation above the mean cooling rate of − 0.057 °C/s found by Li et al. in 2015^2^. The threshold number itself has no intrinsic importance, rather it serves an exploratory purpose and matches the current data by visual inspection. No stratified analyses were conducted until after this threshold was chosen, so that no outcomes would influence where the threshold was set.

### Sub-group analysis

The MIBP ranged from 2.0 to 20.4 s in the faster OSC group and ranged from 3.8 to 100.0 s in the minimal-to-no OSC group. In the stratified analysis, only the faster OSC group showed a significant exponential association between faster OSC rate and shorter MIBP (*p* = 0.001). The minimal-to-no OSC group did not show an association between OSC rate and MIBP (*p* = 0.827).

Table 1 compares subjective responses and clinical outcomes between the faster OSC group and the minimal-to-no OSC group. The stratified groups based on the threshold for OSC did not differ significantly in ocular symptoms as shown by the DEFC score with contact lenses (*p* = 0.897), the DEFC score without contact lenses (*p* = 0.617), the PSQ score (*p* = 0.817), and the ratio of symptomatic to asymptomatic subjects for dry eye among contact lens wearers (*p* = 0.109). The ratio of Asian versus non-Asian ethnicity was also not significantly different between the groups (*p* = 0.710). Sex was statistically significant, with more females in the faster OSC group (89%) than in the minimal-to-no OSC group (59%, *p* = 0.003).

**Table 1:** Comparison of mean (SD) for subjective responses and clinical tests between the faster OSC and minimal-to-no OSC groups.

	Faster OSC N = 55	Minimal-to-No OSC N = 22	*p* value
Age (year)	23.02 (3.32)	23.36 (5.55)	0.737
Sex (% M, % F)	10.9, 89.1	40.9, 59.1	0.003
Ethnicity (% Asian, % Non-Asian)	63.6, 36.4	59.1, 40.9	0.710
MIBP (s)	9.24 (4.05)	27.55 (25.74)	< 0.001
PSQ Score	3.32 (1.44)	3.23 (1.35)	0.817
DEFC without CL	2.50 (1.40)	2.40 (1.30)	0.617
DEFC with CL	2.60 (1.50)	2.10 (1.30)	0.897
SYMPTOM Group (% Asx, % Sx)	42.9, 57.1	71.4, 28.6	0.109
Schirmer I (mm)	15.59 (10.97)	16.41 (10.95)	0.800
NITBUT (s)	8.37 (7.93)	19.76 (21.13)	0.005
TMH (mm)	0.26 (0.08)	0.23 (0.06)	0.156
Tear lipid thickness (nm)	49.05 (14.26)	57.32 (19.05)	0.041
Tear lipid variability (CV)	0.076 (0.029)	0.099 (0.078)	0.066

Clinical test results of Schirmer I test strip wetted lengths (*p* = 0.800), TMH (*p* = 0.156) and tear lipid layer coefficient of variation (*p* = 0.066), a measure of the uniformity of the lipid layer, did not show significant differences between groups. Mean NITBUT was significantly shorter (*p* = 0.005) in the faster OSC group with a mean (SD) of 8.37 (7.9) s, compared with the minimal-to-no OSC group with a mean (SD) of 19.76 (21.1) s. Tear lipid layer thickness also significantly differed (*p* = 0.041), with a thinner tear lipid layer in the faster OSC group with a mean (SD) of 49.1 (14.3) nm, compared with the minimal-to-no OSC group with a mean (SD) of 57.3 (19.1) nm. Multivariable modeling showed that within the faster OSC group, OSC rate and NITBUT were significantly associated with the MIBP (*p* = 0.002). Within the minimal-to-no OSC group, only NITBUT was significantly associated with the MIBP (*p* = 0.035).

## Discussion

Findings here indicate that faster evaporative cooling of the ocular surface is associated with an exponentially shorter amount of time that the eye can be held open without blinking prior to the onset of discomfort for the majority of individuals who exhibit ocular surface cooling between blinks. Increased tear evaporation results in thinner localized areas of the tear film and thus localized tear hyperosmolarity, which activate the inflammation pathway associated with dry eye disease^4, 9^. Inflammation leads to stimulation of polymodal and mechanonociceptor nerve endings and increases activity of cold thermoreceptors to evoke sensations of ocular dryness and pain^26–28^. We propose that by this pathway, faster tear evaporation results in a faster onset of ocular discomfort and shortens the amount of time one can refrain from blinking.

It is apparent in Fig. 5 that a sub-group (28.6%) of subjects exhibited minimal-to-no OSC. The OSC and minimal-to-no OSC sub-groups did not differ significantly in baseline ocular symptoms of dryness with or without contact lenses, pain sensitivity, ethnicity, nor in the ratio of symptomatic to asymptomatic subjects. There were significantly more females than males in the group that exhibited OSC. This difference may be due to higher dry eye prevalence in females than males^29–36^. Sex-associated differences have been noted in the amount of meibomian lipids on the lid margins, greater in males than in females, and in lacrimal gland physiology^37,38^. Females 45 years of age and older have been shown to have a thinner tear film and lower clinically assessed tear film quality than males in the same age category, and than younger females^39^. Additionally, females on average have a faster tear evaporation rate than do males, with the fastest average rate of evaporation in females 45 years and older^40^. These sex-associated differences may explain why there were significantly more females than males among those who exhibited OSC in our study. One limitation of this study is that only minimal information on subject medications was collected for eligibility purposes. A number of medications are known to impact the ocular surface, tear film, and dry eye symptoms, therefore a large discrepancy in medication patterns between females and males could partially explain the difference in gender proportions between the groups who did and did not exhibit OSC. Our study population, however, was generally young and in good health, making gender differences in medication use unlikely to be a significant confounder.

Previous studies have noted Asians to have a longer MIBP^8^, greater tear film instability^29,41^, and more severe and frequent dry eye symptoms^42^ than non-Asians. Li et al. found no difference in OSC rate between Asians and non-Asians (*p* = 0.42)^8^. Our study results were consistent with Li et al., with no significant difference between the OSC sub-groups in the ratio of Asian to non-Asian subjects (*p* = 0.710).

Clinical test results revealed that Schirmer I test strip wetted lengths, TMH, and uniformity of the lipid layer were not significantly different between the groups that did and did not exhibit OSC. Tear lipid layer thickness differed significantly between groups, with a thinner tear lipid layer in the group that exhibited OSC. The presence of a thinner lipid layer in this group is consistent with the hypothesis that tear film evaporation is the initial catalyst for eventual blink onset, as greater lipid layer thickness plays a vital role in inhibiting tear evaporation^43,44^. This was further confirmed by the fact that although lipid layer thickness and NITBUT were both significantly related to OSC in the faster OSC group, when NITBUT was included in a multivariable model, lipid layer thickness was no longer significant. This occurs when two explanatory variables are highly collinear, and a significant correlation between a thin lipid layer and faster tear breakup has been documented many times in the literature^45–48^.

The two sub-groups differed in mean NITBUT, a measurement of tear film instability, with the faster OSC group exhibiting significantly shorter mean NITBUT than the minimal-to-no OSC group. Within the faster OSC group, shorter MIBP was significantly associated with shorter NITBUT and faster OSC rate in the multivariable models. Within the minimal-to-no OSC group, shorter MIBP was significantly associated with shorter NITBUT but not with OSC rate. It has been shown that NITBUT is positively associated with the maximum blink interval, the average of three consecutive readings measured from last blink to when the subject could no longer hold the eyes open, independent of age and gender^49^. Nosch et al. found a strong correlation between shorter NITBUT and a larger OST decrease between blinks while watching a video, as well as between shorter NITBUT and increased blink rate^50^. Tear film instability plays an important role in the cycle of evaporative dry eye disease along with tear hyperosmolarity, apoptosis, and inflammation, and is integral to the definition of dry eye disease^51,52^. Our findings of significant associations between shorter MIBP and shorter NITBUT in both sub-groups of subjects suggest that tear film instability plays a role in the pathway that leads to the onset of discomfort and the stimulus to blinking.

Subjects were asked to hold their eyes open until onset of ocular discomfort. Both corneal nociceptors and cold thermoreceptors may activate to signal ocular discomfort, contributing to the need to blink. Ocular surface pain recognition is activated by polymodal and mechanonociceptor neurons, while ocular surface cooling recognition is activated by cold thermoreceptors^26^. Polymodal nociceptors make up approximately 70 percent of corneal nociceptors and activate in response to mechanical, chemical, and warming thermal stimuli^26,27,53^. Mechanonociceptors make up approximately 20 percent of corneal nociceptors and activate in response to purely mechanical stimuli. Constituting approximately 10 percent of the total corneal sensory nerve supply, cold thermoreceptors activate in response to cold air or cold probe stimuli as well as to menthol and sucrose chemical solutions, and are known to play a role in ocular discomfort and the stimulus to blinking^28,53–56^. Studies have demonstrated that activation of corneal cold thermoreceptors induces a blink reflex^54,55^. In a small sample size study Acosta et al. found that cooling of the cornea by 1–2 °C with moderate air temperatures using a gas esthesiometer almost exclusively activated cold thermoreceptors to evoke a sense of cooling^56^. Cooling the cornea by 5 °C from basal corneal temperature also induced sensations of irritation and pain. Our study was performed under near uniform temperature and humidity conditions with continuous low air flow from normal examination room ventilation. Under comfortable environmental conditions, it has been shown that cold thermoreceptors contribute to stimulating basal tear production and spontaneous blinking, but are unlikely to activate a conscious sensation of dryness or cooling^54^. Although sensory nerve activation was not measured directly in this study, our findings suggest that the pathway of increased tear evaporation leading to increased tear osmolarity and inflammation primarily activates polymodal nociceptors to signal ocular discomfort and the need to blink.

While OSC may contribute to sensations of discomfort that lead to blinking for most people, OSC may not be the only mechanism responsible for the onset of blink as demonstrated by the subset of subjects with minimal-to-no OSC that nevertheless had a short MIBP. It has been shown that various changes to ocular surface conditions affect blink rate. Decreased maximum blink interval is associated with increased ocular surface area exposure to the environment, and increased maximum blink interval is associated with topical anesthesia and artificial tear application^57^. Increased corneal sensitivity, as measured with an air pulse stimulus, has been shown to be significantly associated with increased blink frequency^50^. Additionally, pain perception plays an important role in how long one can hold the eyes open comfortably before needing to blink. Li and Lin studied the relationship between the MIBP and pain sensitivity and found that subjects who were able to hold their eyes open longer comfortably had lower average pain sensitivity as measured by the PSQ^8^. Anxiety and stress have also been shown to increase blink rate^58,59^. Awareness of being observed or recorded may alter a subject’s behavior as defined by the Hawthorne Effect and may increase blink rate, particularly within the first minute of observation^60^. Subjects in this study were asked to fixate on the center of the thermal camera lens while an investigator recorded the MIBP and OST. Although the target of fixation and calm manner in which instructions were given for the task are unlikely to have induced stress, it is possible some subjects experienced stress or anxiety related to completing the task. The presence of an instrument in close proximity to the subject’s eyes, keeping one’s head fixed, the experience of a new procedure, or knowledge of being recorded may unintentionally cause a change in behavior that contributed to a shorter interblink period. There are numerous mechanisms which may have caused this subset of subjects to blink before OSC could be observed, so it is unknown if these subjects would demonstrate OSC if there were no other factors leading the subject to blink.

Two subjects exhibited a slight OST increase during the MIBP. One subject exhibited a slight warming throughout the interblink period; the other subject exhibited an initial period of OSC followed by a period of slight warming. Li and Braun formulated a model of the human tear film, evaluating OST changes during the interblink period, and found that in order for the tear film to decrease in temperature, energy loss due to tear evaporation must be greater than the energy provided from the aqueous humor^61^. That is, if the evaporative cooling is small and heat provided from the aqueous humor is large, a slight increase in OST is possible. This may explain the slight monotonic increase in OST during the MIBP exhibited by that subject. Additionally, Li and Braun’s model predicted an OST decline during the initial 20 s for thinning rates of 12 μm/min and 20 μm/min, but then an increase to reach a steady state value when tear evaporation stops. The stabilization of temperature upon cessation of tear evaporation may explain why a single subject initially demonstrated OSC followed by a period of slight warming. It has been shown that the nasal conjunctiva exhibits higher temperature than the temporal conjunctiva and central cornea^62^. Thus, it is possible that in defining the area of interest on the thermographic images for these 2 subjects a region of nasal conjunctiva was included in the demarcated area used to estimate the cornea-averaged temperature, leading to a measurement of ocular surface warming. Terada et al. measured corneal and eyelid temperatures in both control subjects and subjects with Meibomian gland dysfunction, and recorded eyelid temperatures 1–2 °C warmer than corneal temperatures^63^. It is also possible that frames demarcating the area of interest included a small section of the warmer upper eyelid, contributing to a measurement of apparent OST warming. This may occur if a subject was unable to keep the eyes open to maintain a consistent palpebral aperture size or partially blinked during the recording.

Measurements made in this study evaluated the average temperature of all pixels in a defined region of interest as a function of time. By averaging OST across the area of interest in each frame, we do not directly assess how local differences in OSC rate may affect the MIBP and the onset of ocular discomfort. Begley et al. observed regional and global fluorescein tear film thinning and breakup and their associations with ocular discomfort^10^. In both groups they found the maximum blink interval to be inversely associated with rate of increasing discomfort, and a sharp increase in discomfort with tear breakup. The mathematical model of Peng et al. also predicted that localized tear breakup areas experience significantly higher osmolarity than that of non-breakup areas^64^. These studies suggest that localized regions of breakup will lead to discomfort and may influence the MIBP. While averaging the temperature over the region of interest does not highlight localized differences in OSC rate, it does not mask the changes to OST over time and their association with ocular discomfort, as our region of interest includes both tear film breakup and non-breakup areas. Further research into localizing OST measurements to smaller regions of the cornea, and studying the relationships between local temperature changes and onset of ocular discomfort is ongoing.

In summary, faster OSC is associated with an exponentially shorter maximum interblink period for the majority of the study cohort. This is likely because evaporative cooling of the ocular surface is a sign of tear thinning and localized increases in osmolarity that stimulate corneal nerves and initiate the onset of ocular discomfort and the need to blink. The presence of a subset of subjects with no or minimal OSC indicates that evaporative cooling is not the only mechanism responsible for the onset of ocular discomfort. Tear film instability is also clearly a part of the mechanism that begins with aqueous evaporation and culminates in ocular discomfort and the need to blink.
